# Durability of Accoya Wood in Ground Stake Testing after 10 Years of Exposure in Greece

**DOI:** 10.3390/polym12081638

**Published:** 2020-07-23

**Authors:** George I. Mantanis, Charalampos Lykidis, Antonios N. Papadopoulos

**Affiliations:** 1 Department of Forestry, Wood Sciences and Design, University of Thessaly, GR-43100 Karditsa, Greece; mantanis@uth.gr (G.I.M.); lykidis@uth.gr (C.L.); 2 Department of Forest and Natural Environment Sciences, International Hellenic University, GR-66100 Drama, Greece

**Keywords:** acetylation, Accoya wood, durability, decay, stake test, mechanical properties

## Abstract

In this research, acetylated wood (Accoya) was tested in ground contact in central Greece. After ten years of exposure during a ground stake test, acetylated pine wood (*Pinus radiata*) stakes, with a 20% acetyl weight gain, were completely intact and showed no visual decay (decay rating: 0). However, the key mechanical properties of Accoya wood, that is, modulus of elasticity (MOE) and modulus of rupture (MOR) after 10 years of ground contact, were significantly reduced by 32.8% and 29.6%, respectively, despite an excellent visual result since no evidence of fungal attack was identified. This contradiction could possibly indicate that the hallmarks of decay, i.e., brown-rot decay of acetylated wood can be the significant loss of mechanical properties before decay is actually visible.

## 1. Introduction

**Figure 1:**
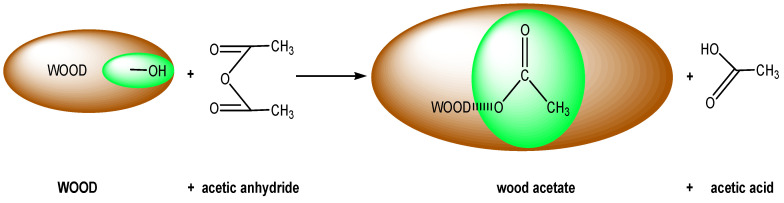
The reaction of wood with acetic anhydride [6].

The main target of applying chemical modifications in wood is to provide dimensional stability and decay resistance [1,2,3,4,5,6]. Researchers have mostly studied the reaction of hydroxyl groups with acetic anhydride, a process that is called acetylation. The reaction is depicted in Figure 1, where the wood hydroxyl groups are replaced by the acetyl groups of the acetic anhydride and the wood remains in a swollen condition due to the bulking action of the acetyl groups within the cell walls. 

The first attempt of producing acetylated wood has been recorded in 1865, when Schutzenberger acetylated cellulose. Fuchs [7] and Horn [8], in the late 1920s, were the first researchers who acetylated wood flour; whole wood was firstly acetylated by Stamm and Tarkow [9]. Since then, wood has been successfully acetylated in a variety of ways by many laboratories worldwide [10,11].

Acetylation appears suited to provide adequate protection against biological attack for materials derived from non-durable wood species [12]. Dr. Harold Tarkow at the Forest Products Laboratory in Madison, Wisconsin, was the first scientist to demonstrate the high resistance of acetylated wood, namely, balsa, to fungal decay [13]. The protection threshold levels for acetylated pine was determined by Beckers et al. [14]. They found that a weight gain of 18% was required against the brown-rot fungi, and a value over 20% was required against the white-rot fungi. A value of 20% was reported to prevent an attack by fungi in the ground contact stake tests [15]. Suttie et al. [16] reported that the bulking effect is the only factor that determines the protection from decay. Hill and Papadopoulos [17] reaffirmed this conclusion when they presented a comprehensive evaluation of the effectiveness of linear chain anhydrides against brown-rot fungi. This conclusion was further reaffirmed when pine samples were exposed to soft-rot decay [18,19]. Few studies have been published so far that focused on the protection against invertebrates [20], including marine wood borers. Papadopoulos et al. [21] used a laboratory test, which was developed by Borges et al. [22], and assessed if the modification of pine wood with a series of anhydrides offers resistance to the crustacean wood borer *Limnoria.* The results revealed, firstly, that modification resulted in a significant reduction in the number of fecal pellets produced and, secondly, that the anhydride type had a minimal effect on feeding. A recent study has shown that pine wood modified with a series of anhydrides afforded significant protection in feeding above a 16% level of modification against the subterranean termites [23]. After 1995, a series of field trial results has been published [24]. Still, results from long-term field trials are sparse, although Larsson-Brelid and Westin [25] reported that the resistance of acetylated wood (with an acetyl content of ca. 20%) to fungal decay after 18 years in soil contact was equivalent to CCA-treated wood (Chromated Copper Arsenate) at a high retention level (e.g., 103 kg/m^3^). Based on the surveyed literature, it seems that the lowering of the equilibrium moisture content in combination with the degree of cell wall bulking is the primary mode of action [6,12,26,27,28,29,30,31,32,33].

A recent review by Ringman et al. suggested that decay resistance in modified wood is caused by a reduced wood moisture content (MC) that inhibits the diffusion of oxidative fungal metabolites. It has been reported that a MC below 23–25% will protect wood from decay, which correlates with the weight percent gain (WPG) level revealed to inhibit decay in modified wood for several different ways of wood modification [34]. Recent studies have shown that modified wood is not permanently protected from brown-rot decay even at high modification levels [35,36,37]. Current literature indicates that the wood modification itself may initially be degraded, at least locally, generating areas of sufficiently low modification levels to allow for diffusion/degradation [37,38,39]. More long-term degradation experiments with modified wood are needed to confirm this theory, but the potential implications are large. A better understanding of how degradation eventually occurs in modified wood will help improve the existing modification procedures and guide the development of future, environmentally friendly wood protection systems.

The first attempt to commercialize the process was not successful in the USA (1961), Russia (1977), and Japan (1984) [6]. On a semi-industrial level, the first successful scaled-up acetylation was performed at Stichting Hout Research, in the Netherlands, by Prof. Holger Militz and coworkers [40]. Most of the commercial developments of modified wood have taken place since 2006, and Hill [41] gives an excellent update on the recent commercial status. Commercial production started with Titanwood in the Netherlands in 2007, and the Eastman Chemical Company commercialized the product in the USA in February, 2012 [42]. Presently, acetylated wood is industrially produced by Accsys Technologies in Arnhem, the Netherlands. It is marketed today under the commercial name Accoya, utilizing predominantly radiata pine wood (*Pinus radiata* D. Don), and to a lesser extent, beech (*Fagus sylvatica* L.) and alder (*Alnus* sp.), having a 20% acetyl weight gain on the average [42]. Nowadays, Accoya wood is available worldwide, and its main uses are typically in the outdoors, above or in ground contact. 

The aim of this research was to report on the performance of Accoya wood during a ground stake test in Greece after ten years of testing. Such studies are both of scientific and practical importance because they demonstrate the performance of the material in real conditions; however, they are limited in the literature.

## 2. Materials and Methods

**Figure 2:**
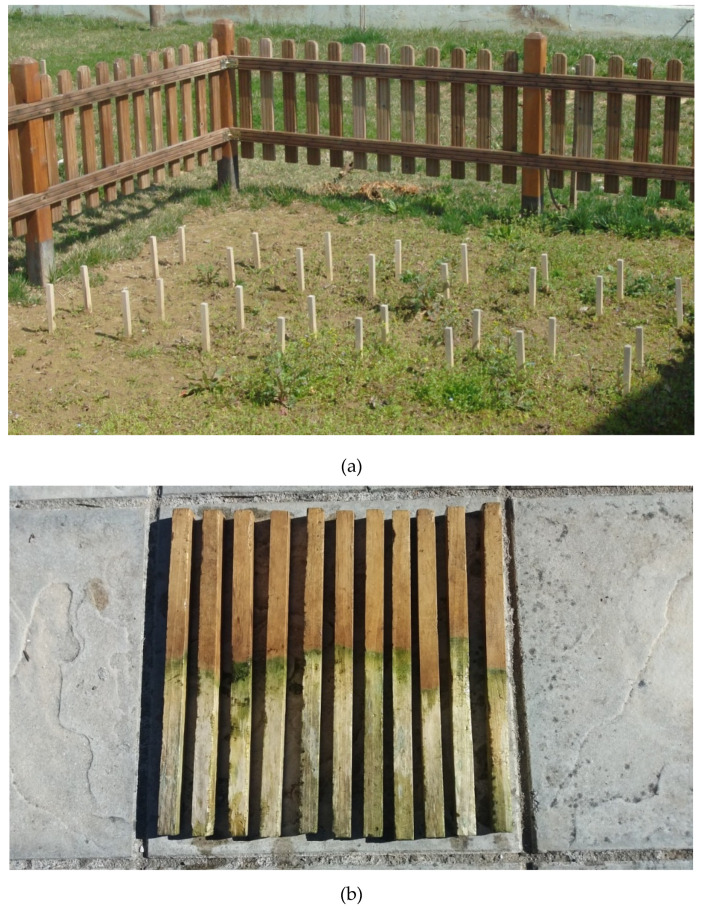
Soil test area used in the experiment (**a**), Accoya stakes as removed after 10 years of ground stake testing in Karditsa, Greece (**b**).

The test site in Karditsa, Greece (39°22′21.7″ N, 21°54′29.6″ E), was considered as the soil test field (Figure 2a). The soils of the area are heavy clay soils, rich in Fe and Mn oxides, sufficient in vital minerals, fertile but not productive due to bad aeration and cohesiveness. They belong to the Alfisols, Entisols, and Inceptisols order of Soil Taxonomy. The soils of the study area have developed from limestone and are classified as Xeralfs according to Soil Taxonomy. The A horizons have clay loam textures (% Sand:% Silt:% Clay 30:36:34 for the Forest surface horizon, and 26:38:36 for the Ap horizon at agricultural land, mean pH (soil:water 1:2.5) is 7.82, an organic matter content of 1.9% for the cultivated plots, and 2.4% for forest soils. The CaCO_3_ levels were found 1–7% at the forest sites and 1–14% at the cultivated plots. This type of soil warrants degradation of the modified wood materials [43].

A parameter, ‘*the climate index value*’, was proposed by Scheffer [44] to estimate decay hazard by geographic location within the conterminous United States for wood exposed above ground to exterior conditions. The climate index value has become widely recognized and is commonly termed the ‘*Scheffer index*’. The index value may be calculated from local weather data to estimate the local decay hazard that existed over a specified time period. The index value has been used in this way to estimate decay hazard that existed during field studies. An index less than 35 represents the least favorable conditions for decay; 30 to 65, intermediately favorable conditions; and greater than 65, conditions most conducive to decay. The Scheffer index for the test site in Karditsa, Greece, was calculated to be 19.8. As a metric by which a relative hazard can be compared between geographic locations, the Scheffer index is not intended to predict the decay propagation rate nor time to failure in specific constructions. Recently, Brischke and Rapp [45] reported that wood temperature and moisture content better predicted decay than climate conditions, as expressed by the Scheffer index. Their findings would be logically expected in as much as decay propagation has been recognized for decades as dependent on moisture and temperature conditions in the wood substrate. 

Accoya wood was supplied by Accsys Technologies S.A. in Arnhem, the Netherlands. Forty-eight acetylated wood specimens, each measuring 360 mm × 20 mm × 20 mm, were conditioned, measured, and placed vertically in the soil. The stakes were placed in such a way that half of the stakes were below the ground line. Each stake was rated below the ground line and thickness was measured at the ground line. Stakes were rated according to the American standard ASTM D1758-02 as follows: a rating of 10 means no decay, 9 means slight decay, 7 means moderate decay, 4 means severe decay, and 0 means total decay [46]. In addition, untreated European pine (*Pinus sylvestris* L.) wood samples were used as controls.

Bending strength, that is, modulus of rupture (MOR), as well as bending modulus of elasticity (MOE) of the acetylated specimens were evaluated in the beginning of the test, at 1-year time, and at 10-year time according to standard DIN 52186 [47]. The experiment is still ongoing. Thus, every time, MOE was determined using ten specimens measuring 360 mm × 20 mm × 20 mm. The MOE test was performed on a universal Zwick-Roell Z020 universal testing machine (Zwick-Roell, Kennesaw, GA, USA) once specimens reached a constant weight at 65% relative humidity and 20 °C temperature, using three-point static bending, where growth rings were horizontally orientated in accordance with DIN 52186. The load (F) was applied at a constant rate of 10 mm/min. Similarly, MOR was estimated during the same test using the maximum load (F_max_) at the breakpoint as the condition of rupture. 

Data from the mechanical properties measurements were analyzed using a one-way analysis of variance (ANOVA) (α = 0.05) using the SAS software (version 9.2). Differences between individual treatments were analyzed using Duncan’s multiple range test using SPSS/18. 

## 3. Results and Discussion

Table 1 demonstrates the performance of Accoya stakes during the testing period and Figure 2b depicts their appearance after ten years of ground contact. Control stakes were relatively sound (slight decay) after the first twelve months of testing and were totally decayed after five years of testing. After ten years, Accoya wood stakes were still completely intact and have shown no decay (decay rating: 0), according to the American standard ASTM D1758-02 [46].

As clearly shown in Table 2, the mechanical property measuremets of acetylated wood specimens decreased over time. However, this reduction was not statistically significant after the first year of testing. MOE and MOR values of Accoya wood stakes, over the 10 years of testing, were reduced by approximately 32.8% and 29.6%, respectively, as compared with the respective values of acetylated wood before the start of the test (Table 2). 

Scion, formerly known as the New Zealand Forest Research Institute, examined the durability of Accoya wood in a field stake test and compared its performance to naturally durable species (teak, merbau, cypress, and cedar) and preservative-treated wood. Accoya wood showed minor decay (0–3% of the cross section—decay rating: 9) after ten years of testing and performed better than naturally durable species, and even better than preservative-treated wood (Chromated Copper Arsenate) with CCA-H3.2 (hazard class rating: 3.2, outside, above ground, uncoated horizontal) and CCA-H4 (hazard class rating: 4, outside, in ground contact) [48]. This study was not published, and after an exhaustive search, we were not able to find the full report.

A test conducted by the Environmental Research Centre (Naresuan University, Thailand), involved setting up ground stake tests at sites around Thailand. After 5-year exposure, Accoya wood lightly established decay (3–10% of the cross section—decay rating: 8) and performed much better than high-quality teak and mahka wood [48].

From the above discussion, it becomes clear that the decay rating of Accoya wood in a Mediterranean country like Greece is zero (0) even after ten years of testing, whereas in New Zealand, the decay rating is nine (9) after ten years of testing, and eight (8) in Thailand after five years of testing. At this point, in the lower part of Table 1, data from acetylated OSB (Oriented Strand Board) in field tests performed in Greece [49], and data from acetylated fiberboards in field tests performed in Mississippi (USA) and Indonesia were also included for comparable purposes [50]. This set of data reaffirms the above observation. Furthermore, under the same framework, Larsson-Brelid and Westin [25] addressed the resistance to biological degradation of acetylated wood in Sweden and Finland. Results from this test were presented after five years of exposure and showed that the resistance of acetylated wood with an acetyl content of about 20% was in the same range as that of wood treated to the high retention of the reference CCA preservatives. At acetylation levels above 20%, none of the samples were rated higher than 1, neither in Sweden nor in Finland. After 12 years of exposure, the average index of decay was 51 in Sweden and 45 in Finland for the medium level of acetylation (average acetyl content: 19.8%), compared to the high retention level CCA preservative, where the index of decay was 41 and 31, respectively. Making the same comparison after 18 years of exposure in Sweden, the index of decay was 56. For the acetylated test stakes, the corresponding figures were 65 for the medium level and 60 for the high acetylation level (average acetyl content: 22%). This implicated that in order to be comparable with the CCA preservative for 18 years, an acetyl content of above 22% is needed. Westin et al. [51] also tested the marine borer resistance of acetylated wood. The testing, according to the European standard EN 275, was done in a bay on the Swedish west coast. The marine borer (mainly *Teredo navalis*) activity at the test site is very high, always resulting in failure of control specimens within a year. It was found that specimens of acetylated Scots pine (WPG, 21%) was severely attacked after 16 years, and acetylated southern yellow pine specimens at the higher acetylation level were either completely sound or only slightly attacked after 11 years of exposure, whereas specimens at the lower acetylation level all failed in three years.

It has been reported earlier that the Greek field is slower in terms of decay compared to other territories, for instance, in central and northern Europe, or in tropical regions [49]. The Karditsa area has a very dry, desert-like, long summer season (May–Oct.), with temperatures ranging up to 30–40 °C, and also a short, wet winter season (December–March) with mostly rains and a high relative humidity (>80%) and mean temperatures between 0 and 12 °C, very rarely below zero. The average annual rainfall during the test period was approximately 680 mm.

**Figure 3:**
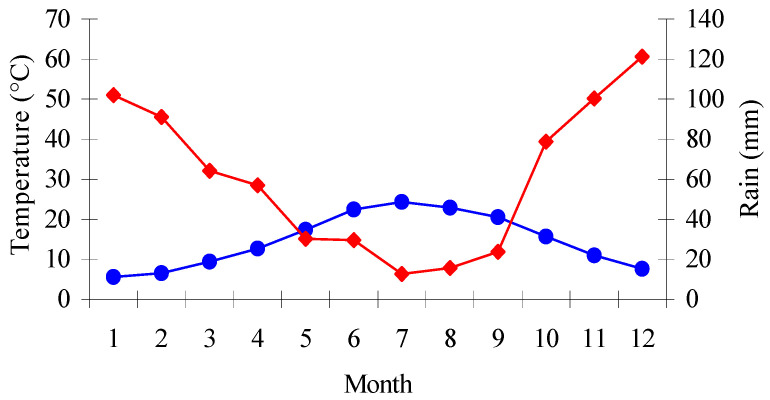
Ombrothermic diagram: mean temperature of air (●), mean monthly precipitation (■). The period in which the mean monthly precipitation is below the mean temperature of air denotes the dry season.

Based on the ombrothermic diagram in the Karditsa area (Figure 3), the dry periods in the exposure site were very long, up to six months (May–October) every year, and this itself had an inhibitory effect on fungal growth [49,52,53]. It is well-known that a long dry period results in a significant reduction of fungal activity [49,50]. It is likely that presence of the soft- and white-rot decay in the samples is related with the moisture of the soil. The change in soil moisture affects the oxygen level in the ground [43]. Moisture variation influences the moisture content in planted wood specimens in the field soil, and changing the moisture of wood consequently and directly affects the oxygen level in the wood [54,55]. Therefore, oxygen is a crucial factor which has a beneficial effect on the various activities of the microorganisms in wood [52,53,54]. In conclusion, it is apparent that the typical Greek climatic conditions, like the dry ones (Karditsa area) in a south Mediterranean dry zone, are much less harsh on wood, as compared with the climatic conditions in central and northern Europe.

Based on the above discussion, the two main conclusions that can be drawn were, firstly, that acetylated wood (Accoya) was completely intact and showed no decay (decay rating: 0) after ten years of exposure during the ground stake test, and, secondly, that its key mechanical properties, MOE and MOR, were significantly reduced by 32.8% and 29.6%, respectively. However, this contradiction between visual and mechanical tests, or in other words, the combination of low climate risk with respect to visual inspection and the significant loss of mechanical properties, requires further explanation. 

In a key study on the resistance of acetylated wood to biological degradation in ground contact, the stakes were removed from the field and analyzed for acetyl content [15]. It was reported that after two years of testing, no significant differences were observed in acetyl content on the part of the stake that was exposed above ground level. However, the part of the stake that was below ground level had a much lower acetyl content, with a drop of approximately 30%. This lower acetyl content in the part that was below ground level was attributed to the hydrolysis of the ester bonds and/or to microbiological activity. This also seemed to be the case in this study. 

Furthermore, it is likely that visual inspections of acetylated wood suffer the same limitation as untreated wood: incipient decay resulting in significant mechanical properties loss may go undetected by visual inspection. If the incipient decay is brown-rot (likely because this is a pine), Fenton chemistry is their hallmark. Fenton depolymerizes the carbohydrates and rearranges the lignin. Depolymerization of the carbohydrates, particularly cellulose, is typically credited for the strength losses [56]. In addition, the small decrease in density (~5%) after 120 months of testing, as depicted in Table 2, may correspond to a loss in mechanical properties.

## 4. Conclusions

This research is a presentation of results obtained after a 10-year ground stake test in Greece in which acetylated wood (Accoya) was tested in a typical southern Mediterranean zone. It was shown that even after 10 years of ground contact, Accoya wood performed very well, with no visual decay signs. It also maintained its structural integrity to some extent; although, the MOE and MOR properties of Accoya wood were decreased to a considerable extent. This contradiction could possibly indicate that the hallmarks of decay, i.e., brown-rot decay, can be the significant loss of mechanical properties before decay is actually visible.

## Figures and Tables

**Table 1:** Decay rating of acetylated wood in field tests.

Decay Rating *		
Exposure Time (In Months)	Control	Accoya Wood (Acetyl Weight Gain ~20%)
0	10	10
12	9	10
60	0	10
120	-	10
Data from acetylated OSB in field tests performed in Greece [49]		
48	0	10
72	-	7
96	-	4
102	-	0
Data from acetylated fiberboards in field tests performed in Mississippi [50]		
12	4	10
32	0	9
Data from acetylated fiberboards in field tests performed in Indonesia [50]		
1	7	10
12	0	4

* A rating of 10 means no decay, 9 means slight decay, 7 means moderate decay, 4 means severe decay, and 0 means total decay. Mean values of 10 replicates.

**Table 2:** Mechanical properties of Accoya wood in ground stake test in Greece.

Exposure Time (In Months)	Mean Density (g/cm^3^)	Mechanical Properties *	
		MOE (MPa)	MOR (MPa)
0	0.537 (0.042)	9.530 (1.400) a	96.8 (18.5) a
12	0.522 (0.029)	9.460 (1.330) a	92 (11) a
120	0.508 (0.021)	6.400 (475) b	68.1 (7.5) b

* Mean values of 10 replicates (standard deviations in the parentheses). Values followed by the same letter for a given property do not differ from one another by Duncan’s multiple range test (α = 0.05).
